# On the Employment of Chlorate of Potass

**Published:** 1857-01

**Authors:** 


*On the Employment of Chlorate of Potass.* By M. Isambert.

In this paper M. Isambert, after giving the history of the employment of the chlorate since its discovery by Berthollet, its disuse, and recent revival by Hunt and others, state that he has of late investigated its therapeutical action in M. Blache's wards, at the Children's hospital, and its physiological effects, by experiments upon himself. Passing over these latter, we briefly present the conclusions he has arrived at in regard to its medicinal employment.

1. *Gangrene of the mouth.*—On carefully examining Mr. Hunt's observations, he considers it very doubtful whether he has always had to do with true gangrene of the mouth, having rather confounded this affection with ulcero-membranous stomatitis, in which the effects of the chlorate are truly remarkable. In two cases of gangrene he did not find it very serviceable; and West, who carefully distinguishes between the two affections, seems to have come to the same conclusion.

2. *Ulcero-membranous stomatitis.*—This term, adopted by Rilliet and Barthez, well explains the nature of the affection, there being, in fact, both ulceration and the formation of false membrane present, the one predominating in some cases, and vice versa. It is a most obstinate affection, having no natural tendency to a cure, and being liable to a relapse. West first employed the chlorate in this affection, and his success with it has been amply confirmed by Blache, Herpin, Bergeron, and others on the continent. Eight cases which have occurred to the author speak equally favorable. Relapse may, however, occur, though far seldomer than under any other remedy; and it should, therefore, be continued for some time after the fall of the false membrane. The chlorate, too, is powerless against the alveolo-dental pyorrhœa, or ulceration of the borders of the gum, with purulent issue from the alveoli on pressure being made upon the gum. The mean duration of treatment of these eight cases was from three to five days for the production of the fall of the membrane, and five to ten for a complete cure. When the cure was longer delayed, relapse had occurred, or the alveolo-dental pyorrhœa was present.

3. *Aphtha.*—The vesiculo-ulcerative state of the buccal cavity, to which this appellation is now confined, is in general a very mild affection, and curable by simple means. Sometimes, however, numerous and confluent ulcers produce much pain, impede feeding, are very tedious in healing, and induce constitutional disturbance. In a case of this kind the chlorate effected a rapid cure.

4. *Muguet.*—M. Legroux has tried it in several cases of epidemic muguet at the Hotel-Dieu, but without any favorable result. During the trials it was found to pass rapidly into the milk of the nurses, and in this way it may be administered to infants.

5. *Scorbutus.*—M. Fremy has found the medicine of use in this diseasc; and thus we find the moderns returning by another route to one of the first affections the chlorate was recommended for, on the theory of deoxidizing the salt in the economy.

6. *Diptheritis.*—Observations commenced by M. Blache, and continued by the author, leave no doubt as to the utility of the chlorate. In this affection there is, however, every gradation from the most simple to the most malignant form, a sign of most unfavorable augury being found in the swelling of the parotid and deep-seated cervical glands—enlargement of the submaxillary glands occurring in even the simplest forms. Of thirteen cases, the chlorate was exclusively employed in four, and the cure was rapid, the cases being mild ones. In two, although cauterization, with nitrate of silver, was employed at the beginning, the success attributable to the chlorate. In two others, cauterization was simultaneously employed, but the cure was not more rapid than in the others. The ninth case was a very severe one following scarlatina, and the patient was cured by the chlorate and quinine, without the aid of cauterization. The four others died, but they were cases of a very grave description. The chlorate is, therefore, no heroic remedy, always curing ingina miligna, nor is its action immediate; for, although it appears in the saliva a few minutes after administration, it requires at least twenty-four hours, and usually three or four days, before it can effect its purpose. It should, therefore, be commenced with early.

7. *Croup.*—The success attendant upon the chlorate in diphtheritis naturally led to its employment in croup. The author relates four cases in which the chlorate seemed to have succeeded, and refers to eleven others, in which tracheotomy was resorted to also, whether because the medicine did not seem to be taking effect with sufficient speed, or that tracheotomy having been already employed, it was given as an adjuvant to prevent the reproduction and extension of the diphtheritis. Of these eleven cases, some of which were very severe, there were eight recoveries and three deaths. Between the first of January and the end of March, 1856, tracheotomy was performed in M. Blache's wards, fourteen times, with nine recoveries and five deaths, all the children taking the chlorate either prior to or subsequent to the operation. If this success be not due to the occurrence of a run of lucky cases, which occasionally occurs in practice, the result is remarkable, as the proportion of recoveries after tracheotomy, at the same hospital, has averaged during the last six years but one in four to one in five. When tracheotomy has been performed, the use of the chlorate is especially indicated, where there is a tendency in the diphtheritis to extend to the bronchi, pharynx or nasal passages. It should be combined with expectorants, and considerable doses given.*—*Gazette Medicale.*

---

*We publish the observations of M. Isambert, because we are disposed to think that the salts of chlorine are not regarded with sufficient favor by American physicians. The usual dose of the chlorate of potash is from ten grains to two scruples in the 24 hours, either in the form of powder or solution.—*Ed.*

